# Platelet-to-lymphocyte ratio predicts short-term mortality in patients with moderate to severe traumatic brain injury

**DOI:** 10.1038/s41598-022-18242-4

**Published:** 2022-08-17

**Authors:** Wenjuan Li, Wenjing Deng

**Affiliations:** Department of Neuro-Intensive Care Unit, The First Affiliated Hospital of Zhengzhou University, Zhengzhou, Henan China

**Keywords:** Biomarkers, Neurology

## Abstract

An easily accessible biomarker with good diagnostic power for patients with traumatic brain injury (TBI) was needed to predict the short-term mortality. Studies have shown that platelet-to-lymphocyte ratio (PLR) is a biomarker for patients with tumor. This study aimed to identify the relationship between PLR and short-term mortality in patients with moderate to severe TBI. This is a retrospective cohort study. We selected patients with moderate to severe TBI who were admitted to the emergency department of The First Affiliated Hospital of Zhengzhou University. Biomarkers were collected within 24 h after admission. To investigate their relationship with short-term mortality, Cox proportional hazards regression and ROC curve analysis were performed. A total number of 170 patients was included. 47 (27.6%) patients had died and 123 (72.4%) patients were survived by the end of the study. Patients with different Rotterdam CT score (HR = 1.571, 95%CI 1.232–2.002, *p* < 0.001) or PLR levels (HR = 1.523, 95%CI 1.110–2.090, *p* = 0.009) had significant different mortality rates. The AUC curve analysis showed that the AUC of Rotterdam CT score and PLR groups were 0.729 (95%CI 0.638–0.821, *p* < 0.001) and 0.711 (95%CI 0.618–0.803 *p* < 0.001), respectively. PLR level is an independent biomarker with great diagnostic power for short-term mortality in patients with moderate to severe brain injury.

## Introduction

Traumatic brain injury (TBI) has become one of the main causes of death and disability related to traumatic diseases. As of 2016, the number of patients worldwide reached 27 million, an increase of 47% over 1990^1,2^. Traumatic brain injury includes primary injury and secondary injury. The primary injury is mainly caused by mechanical impact, while the mechanism of secondary injury is complicated, which includes a series of injuries such as neuroinflammation, hypoxia, and hypoperfusion. Neuroinflammation is a complicated, non-specific response that can occur in the acute phase of traumatic brain injury. It may last from minutes to years after injury, resulting in persistent neurological damage and poor prognosis^3–5^.

The severity of traumatic brain injury is ranked as mild, moderate, and severe according to the Glasgow Coma Score (GCS). As high as 30–40% of the mortality rate of patients with severe brain injury has been reported^6–10^. A large number of literatures have identified biomarkers for the prognosis of traumatic brain injury. They mainly focus on those in blood and/or cerebrospinal fluid, such as: S100β, GFAP, NSE, UCH-L1, NF, Tau, MBP, etc.^11–17^. However, in the majority of these studies, patients are with either mild or severity-undefined brain injuries. Studies about the prognosis of patients with moderate or severe traumatic brain injury, especially short-term mortality related, are still limited. More importantly, these blood and/or cerebrospinal fluid biomarkers test are hospital grade- and test level-dependent, which tremendously limit their clinical application. The repeated operations of lumbar puncture increase the cost as well as the trauma of patients. Therefore, there is an urgent need for an easily accessible biomarker with better or comparable diagnostic power.

Platelet-To-Lymphocyte ratio (PLR) has good generalizability and could be calculated and obtained by routine laboratory test at admission without further bothering the patients. Studies have shown that PLR was associated with systemic non-specific inflammation, and high PLR implied a poor prognosis for colorectal cancer and non-small cell lung cancer^18–20^. This study identified PLR as an independent biomarker with great diagnostic power for short-term mortality in patients with moderate to severe traumatic brain injury.

## Methods

### Participants

This was a retrospective cohort study. Selected patients with moderate to severe TBI who were admitted to the emergency department of The First Affiliated Hospital of Zhengzhou University from January 2020 to December.

2021, and whose first admission department was Neuro-Intensive Care Unit (NICU). Inclusion criteria: (1) There was a clear history of injury, and the time of injury was within 24 h; (2) Patients with moderate to severe TBI with a GCS score of 3–12 points on admission, severe (GCS score 3–8), moderate (GCS score 9–12); (3) All patients were confirmed by head CT at emergency department. Exclusion criteria: (1) Patients with Abbreviate Injury Score (AIS) ≥ 3 points in organs other than head and neck; (2) Patients also are diagnosed with pregnancy, heart failure, renal failure, tumor, blood system disease; (3) Patients taking drugs that may affect platelet and lymphocyte count and function (antiplatelet aggregation drugs, glucocorticoids, contraceptives etc.); (4) Patients with hospitalization time < 24 h. This study was approved by the ethics committee of The First Affiliated Hospital of Zhengzhou University (2019-KY-220) and in accordance with the Declaration of Helsinki. All patients and their legally authorized representative signed written informed consent on admission.

The outcome of this study was short-term mortality, which referred to 30-day all-cause mortality; the diagnosis of hypertension was defined according to the 2018 ESC/ESH guidelines for the management of hypertension, and the diagnosis of diabetes was defined according to the American Diabetes Association diagnostic criteria. The time of injury was defined as the time from injury to admission. There were four categories of injury, including traffic accident, motorcycle, fall from height, and fall. CT was performed immediately at emergency department, and the CT score was defined by radiologist and NICU specialists according to the Rotterdam CT scoring standard; the GCS score and pupillary were determined by at least two NICU physicians after admission; asymmetry of pupillary was defined as a measurement difference of 5 mm or more in diameter of pupils between two sides; neurosurgery referred to decompression and/or removed of hematoma, ventricular drainage. Blood transfusion included red blood cells, plasma, cryoprecipitate and/or platelets. All patients included in this study did not receive platelet transfusions within 24 h after admission.

### Clinical data

The baseline characteristics of the patients included age, gender, history of hypertension, diabetes, time of injury, categories of injury, Rotterdam CT score,

GCS score, neurosurgery, blood transfusion, the platelet and lymphocyte count within 24 h after admission. Blood was drawn and sent to the emergency laboratory of The First Affiliated Hospital of Zhengzhou University immediately after admission, where Coulter LH750 (Beckman-Coulter company in Calif. USA) was used for testing. GCS score was divided into two groups: moderate (9–12), severe (3–8); PLR = platelet /lymphocyte.

### Data analysis

Continuous variables were shown as mean ± standard deviation. *t* test was performed to compare the difference between survivors and non-survivors. Variance analysis was used to compare different PLR groups; skewed distribution of continuous variables was presented as median (p25, p75), using Mann–Whitney U test for statistics. We presented categorical variables as number and percentage, compared by Chi-square test or Fisher test. Baseline PLR was a continuous variable, divided into four groups according to the interquartile range: PLR group1 (< 128.0), PLR group2 (128.0–207.6), PLR group3 (207.6–330.7), PLR group4 (> 330.7).

Kaplan–Meier survival analysis was used to generate survival curves and we used the Log-rank test to examine the differences between the survival curves. Univariate and multivariate Cox proportional hazards regression models were applied to analyze the association between PLR groups and 30-day mortality by calculating hazard ratios (HR) and 95% confidence intervals (CI). The multivariate Cox proportional hazards regression model included variables which were identified significant association with 30-day mortality in univariate analysis (*p* $<0.05$) or conventional confounding factors (*p* $<0.2$). SPSS 25.0 software was used for data analysis, and *p* < 0.05 was considered statistically significant.

### Ethics approval and consent to participate

This trial has the ethical approval of the ethics committee of The First Affiliated Hospital of Zhengzhou University (2019-KY-220) and in accordance with the Declaration of Helsinki. All patients and their legally authorized representative signed written informed consent on admission.

## Results

### Baseline data

**Figure 1:**
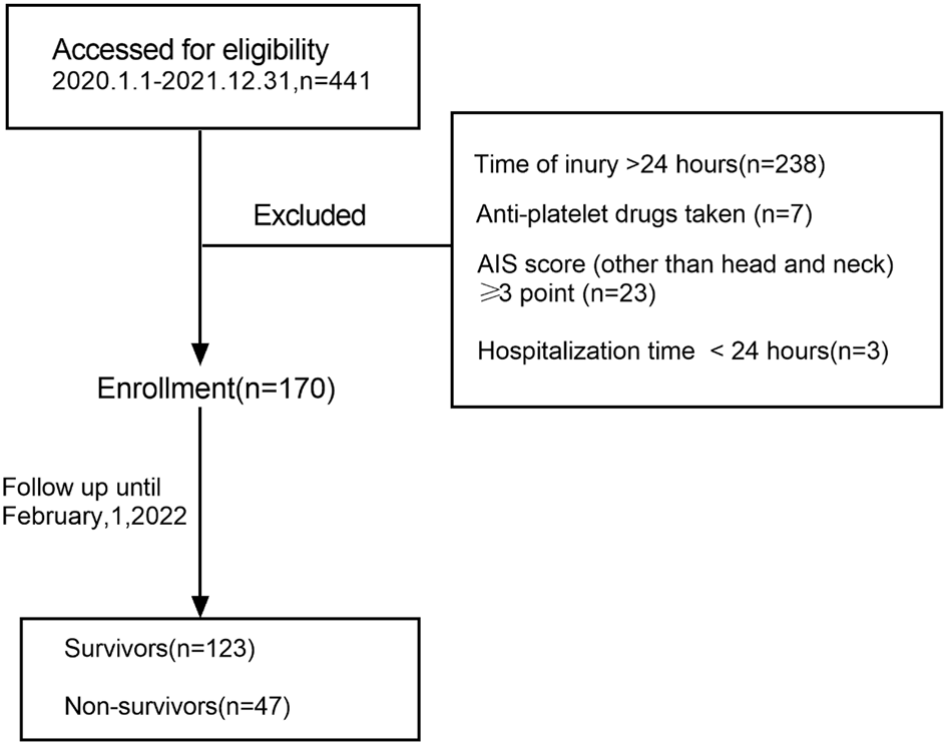
Study flow chart.

The flow chart of this cohort study is shown in Fig. 1. 170 patients were included, with 136 males, accounting for 80%. The mean age was 51.6 ± 15.2 years, median time of injury was 10 (3.8, 22.5) hours, and the main categories of injury was car accident (89, 53%). The mortality of severe TBI was 33.8%, which is consistent with others’ reports. The mortality rate of moderate TBI in our study was 5.4% (Table 1).

**Table 1:** The baseline clinical characteristics of the study cohort.

	All (n = 170)	Survivors (n = 123)	Non-survivors (n = 47)	*p*-value
Age, years	51.6 $\pm$ 15.2	49.6 $\pm 15.2$	56.8 $\pm 14.1$	0.005
**Gender, n (%)**				0.265
Male	136 (80.0)	101 (74.3)	35 (25.7)	
Female	34 (20.0)	22 (64.7)	12 (35.3)	
**Hypertension, n (%)**				0.613
No	138 (81.2)	101 (73.2)	37 (26.8)	
Yes	32 (18.8)	22 (68.8)	10 (31.2)	
**Diabetes, n (%)**				0.062
No	157 (92.4)	117 (74.5)	40 (25.5)	
Yes	13 (7.6)	6 (46.2)	7 (53.8)	
Time of injury, hours	10 (3.9,22.5)	10 (5,24)	8 (2,19)	0.062
**Category of injury, n (%)**				0.754
Car accident	89 (53.0)	65 (73.0)	24 (27.0)	
Motorcycle	16 (9.5)	13 (81.3)	3 (18.7)	
Fall from height	43 (25.6)	31 (72.1)	12 (27.9)	
Fall	20 (11.9)	13 (65.0)	7 (35.0)	
**GCS, n (%)**				0.001
Moderate	37 (21.8)	35 (94.6)	2 (5.4)	
Sever	133 (78.2)	88 (66.2)	45 (33.8)	
**Pupillary, n (%)**				0.865
Symmetry	106 (62.7)	77 (72.6)	29 (27.4)	
Asymmetry	63 (37.3)	45 (71.4)	18 (28.6)	
Rotterdam CT score	170 (2.8 $\pm$ 1.3)	123 (2.5 $\pm 1.1$)	47 (3.7 $\pm 1.4$)	< 0.001
**Neurosurgery, n (%)**				
No	88 (51.8)	67 (76.1)	21 (23.9)	0.253
Yes	82 (48.2)	56 (68.3)	26 (31.7)	
**Blood transfusion, n (%)**				0.047
No	107 (62.9)	83 (77.6)	24 (22.4)	
Yes	63 (37.1)	40 (63.5)	23 (36.5)	
Platelet ($\times {10}^{9}/l$)	180.3 $\pm$ 63.7	185.9 $\pm 59.9$	165.7 $\pm 71.4$	0.065
Lymphocyte ($\times {10}^{9}/l$)	0.8 (0.5, 1.3)	0.7 (0.5, 1.1)	1.0 (0.6, 1.9)	0.01
PLR	207.6 (128.0, 330.7)	232.8 (169.7, 350.9)	124.2 (81.8, 213.3)	< 0.001

To investigate the diagnostic power of PLR in TBI patients, we divided all patients into four groups according to the interquartile range of their PLR level:

PLR group1 (< 128.0), PLR group2 (128.0–207.6), PLR group3 (207.6–330.7), PLR group4 (> 330.7). As shown in in Table 2, from PLR group1 to PLR group4, gradual increase of lymphocyte and decrease of platelet as well as PLR were observed. All the results were statistically significant.

**Table 2:** The clinical characteristics of PLR groups.

	Group1 (n = 42)	Group2 (n = 43)	Group3 (n = 43)	Group4 (n = 42)	*p*-value
Age, years	51.2 $\pm 16.2$	50.9 $\pm 14.7$	52.1 $\pm 15.7$	52.3 $\pm 14.6$	0.967
**Gender, n (%)**					0.124
Male	36 (85.7)	36 (83.7)	29 (67.4)	35 (83.3)	
Female	6 (14.3)	7 (16.3)	14 (32.6)	7 (16.7)	
**Hypertension, n (%)**					0.834
No	35 (83.3)	33 (76.7)	36 (83.7)	34 (81.0)	
Yes	7 (16.7)	10 (23.3)	7 (16.3)	8 (19.0)	
**Diabetes, n (%)**					0.890
No	40 (95.2)	40 (93.0)	39 (90.7)	38 (90.5)	
Yes	2 (4.8)	3 (7.0)	4 (9.3)	4 (9.5)	
Time of injury, hours	10 (7, 17.5)	11 (6, 24)	15 (3, 24)	3 (1.9, 18.3)	0.002
**Category of injury, n (%)**					0.960
Car accident	19 (46.3)	22 (51.2)	23 (53.5)	25 (61.0)	
Motorcycle	4 (9.8)	4 (9.3)	5 (11.6)	3 (7.3)	
Fall from height	12 (29.3)	12 (27.9)	9 (20.9)	10 (24.4)	
Fall	6 (14.6)	5 (11.6)	6 (14.0)	3 (7.3)	
**GCS**					0.034
Moderate	14 (33.3)	10 (23.3)	10 (23.3)	3 (7.1)	
Severe	28 (66.7)	33 (76.7)	33 (76.7)	39 (92.9)	
**Pupillary, n (%)**					0.334
Symmetry	26 (61.9)	28 (65.1)	30 (71.4)	22 (52.4)	
Asymmetry	16 (38.1)	15 (34.9)	12 (28.6)	20 (47.6)	
Rotterdam CT score	2.5 $\pm 1.1$	2.4 $\pm 1.2$	2.9 $\pm 1.2$	3.5 $\pm 1.3$	< 0.001
**Neurosurgery, n (%)**					0.767
No	22 (52.4)	25 (58.1)	21 (48.8)	20 (47.6)	
Yes	20 (47.6)	18 (41.9)	22 (51.2)	22 (52.4)	
**Blood transfusion, n (%)**					0.112
No	30 (71.4)	29 (67.4)	28 (65.1)	20 (47.6)	
Yes	12 (28.6)	14 (32.6)	15 (34.9)	22 (52.4)	
Platelet ($\times {10}^{9}/l$)	202.9 $\pm 50.2$	181.5 $\pm 62.4$	175.3 $\pm 56.6$	161.5 $\pm 77.7$	0.025
Lymphocyte ($\times {10}^{9}/l$)	0.5 (0.4, 0.5)	0.7 (0.5, 0.8)	0.9 (0.8, 1.3)	1.9 (1.2, 2.9)	< 0.001
PLR	412.1 (370.7, 483.9)	255.6 (230.8, 295.2)	176.7 (151.4, 192.4)	86.4 (65.2, 105.7)	< 0.001

### PLR level and clinical outcomes

**Figure 2:**
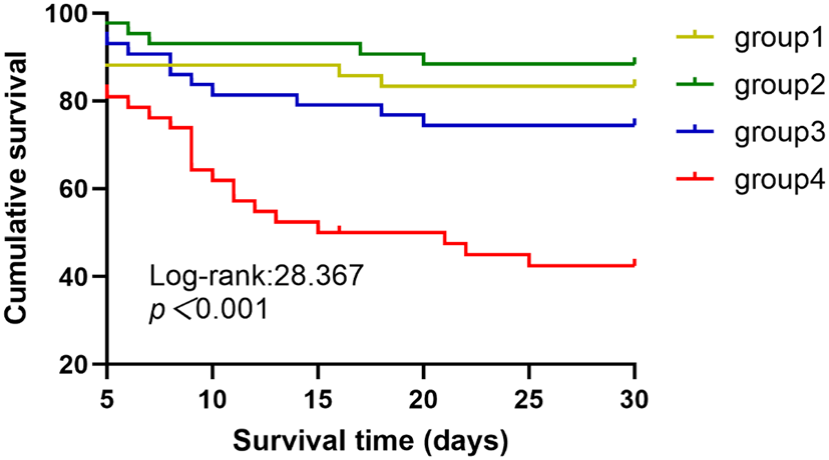
Cumulative risk for PLR groups and mortality in moderate and sever TBI patients. PLR for groups: group1: (< 128.0), group 2 (128.0–207.6), group 3 (207.6–330.7), group 4 (> 330.7).

By the end of the study, 47 (27.6%) patients had died and 123 (72.4%) patients were survived. Compared with the other three groups, group 4 had a shorter median survival time (15, 1.3–28.7), and there was a statistical difference among the four groups (Log-Rank *p* < 0.001) (Fig. 2).

### Association between PLR level and mortality

We applied multivariate Cox proportional hazards regression model to study the association of PLR level with mortality (Table 3). Variables including age, gender, history of diabetes, time of injury, GCS score, Rotterdam CT score, blood transfusion and PLR groups were considered in this model. The results showed that patients with different Rotterdam CT score (HR = 1.571, 95%CI 1.232–2.002, *p* < 0.001) or different PLR levels (HR = 1.523, 95%CI 1.110–2.090, *p* = 0.009) had significant different mortality rates. Importantly, neither platelet nor lymphocyte number alone was as powerful as PLR to predict the mortality of TBI patients (Table 1).

**Table 3:** Association of PLR level with 30-day mortality in univariate and multivariate Cox regression models.

	Unadjusted model		Adjusted model	
	HR (95%CI)	*p*-value	HR (95%CI)	*p*-value
Age	1.028 (1.007–1.050)	0.009	1.023 (0.998–1.048)	0.068
Gender	1.471 (0.764–2.834)	0.249	1.008 (0.494–2.059)	0.982
Diabetes	2.424 (1.084–5.421)	0.031	1.330 (0.557–3.175)	0.521
Time of injury	0.977 (0.944–1.013)	0.205	0.994 (0.961–1.028)	0.713
Rotterdam CT score	1.870 (1.491–2.347)	< 0.001	1.571 (1.232–2.002)	< 0.001
Blood transfusion	1.682 (0.949–2.980)	0.075	0.905 (0.493–1.664)	0.749
GCS score	7.337 (1.779–30.253)	0.006	3.914 (0.910–16.840)	0.067
PLR groups	1.874 (1.394–2.518)	< 0.001	1.523 (1.110–2.090)	0.009

**Figure 3:**
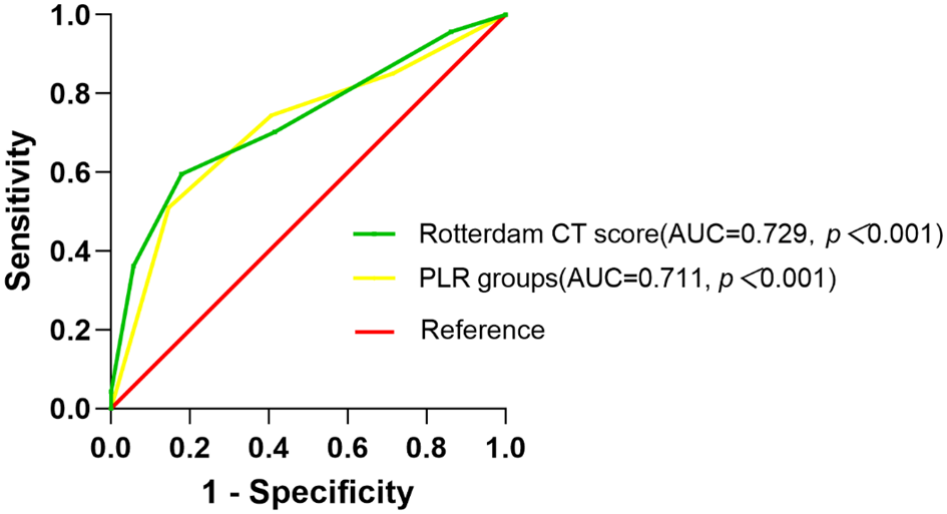
ROC curve of PLR groups and Rotterdam CT score for predicting short-term mortality in moderate to severe TBI patients.

To confirm the diagnostic power of PLR level, we also did ROC curve analysis for short-term mortality. The results showed that the AUC of Rotterdam CT score and PLR groups were 0.729 (95%CI 0.638-0.821, *p*<0.001) and 0.711 (95%CI 0.618-0.803 *p*<0.001), respectively (Fig. 3). The cut-off value was determined to yield largest Youden index. The level of PLR group was 3.5 with sensitivity of 51.1% and specificity of 85.4%, while the cut-off value of Rotterdam CT score was 3.5, with sensitivity of 59.6% and specificity of 82.1%.

All these results suggest that, like the well-known marker Rotterdam CT score, PLR level is an independent risk factor, with comparable diagnostic power, for short-term mortality in moderate to severe TBI patients.

## Discussion

More than 50 million people worldwide suffer from TBI each year, and half of the whole population on earth will experience at least one TBI in their lifetime. However, the treatment and prediction of traumatic brain injury are still big challenges.

The Rotterdam CT score is a method created in 2005 to assess structural brain injury. Compared to the Marshall CT score, Rotterdam CT score added traumatic subarachnoid hemorrhage and intraventricular hemorrhage, simplified the score calculation system, and reduces the assessment difference between emergency department and NICU^21^. Studies have shown that the Rotterdam CT score can predict the mortality of adults and children TBI patients^22–24^, which is consistent with the results in our study.

At present, the biomarker research related to the prognosis of TBI mostly focuses on the combined detection of cerebrospinal fluid and/or blood. Markers such as S-100β, NSE, GFAP, UCH-L1, NF, Tau, and MBP are increased to different degrees in blood and cerebrospinal fluid after TBI, suggesting neuronal and astrocyte damage^11^. The detection of high S-100β in blood and cerebrospinal fluid contribute to identify progressive intracranial hemorrhage after TBI, which is associated with mortality and poor prognosis^15^.

Neuroinflammation is one of the important mechanisms of secondary injury in traumatic brain injury. Studies have shown that a large number of cytokines (TNFα, IL-1β, IL-6, -10, -18 etc.) and chemotaxis (CCL2, 5 and 20, CXCL1, 9 and 10 etc.) can be detected in the cerebrospinal fluid after TBI, which exacerbate oxidative stress and cause persistent neurological damage. For example, IL-18 is associated with disability and cognitive impairment in post-injury patients^25^. However, the detection of these markers has a high requirement for technology and equipment. Furthermore, the samples of cerebrospinal fluid are obtained through lumbar puncture or ventricular drainage. These repeated operations are invasive and not widely accepted by patients and their family members, resulting in limited clinical application and universalness. The aim of this study was to find a convenient and easy-to-follow biomarker for predicting short-term mortality in TBI.

PLR is the ratio of platelet to lymphocyte count, which can be obtained by routine laboratory calculation, and is easy to monitor. Studies have shown that PLR is associated with systemic non-specific inflammation and can predict poor prognosis of variety diseases, especially tumors, heart failure, etc.^19,20,26^. Different cut-off levels of PLR have been reported in patients with NSCLC, colorectal cancer and gastric cancer, varying from 150, 160 to 235 ^27–29^. Despite the actual value, high pretreatment PLR suggests poor overall survival and progression-free survival in cancer patients, which is a reverse trend for TBI patients in our study. High PLR is also related to all-cause mortality in peritoneal dialysis patients, which has been reported recently^30^.

To our knowledge, this is the first research studying the relationship of PLR level with short-term mortality in TBI patients with moderate to severe TBI. Low PLR is mainly achieved by decreasing platelet count and increasing lymphocyte count. The primary injury mechanism of TBI leads to the rupture of capillaries and vessels and the destruction of the blood–brain barrier (BBB), triggering the interaction between platelets and endothelial cells or subendothelial matrix. This results in platelet adhesion-activation, and the formation of platelet embolism at the injury site for hemostasis. The balance between coagulation and anticoagulation is broken in moderate to severe TBI patients, leading to platelet overactivation and the number decreases at the early stage of injury. The spontaneous aggregation and subsequent excessive consumption induce secondary platelet depletion and increase bleeding risk. Studies had shown the increasing risk of intracranial hemorrhage progression when the platelet was less than 175 × 10^9^/L^31^, and nine-fold higher mortality when the number is below 100 × 10^9^/L^32^.

Huang et al. reported the effect of different gender on PLR ratio to predict cardiovascular mortality of peritoneal dialysis (PD) patients. For female PD patients, high PLR ratio was related to worse prognosis due to higher level of estrogen and low levels of serum iron^33^. Our study did not find the same effect of gender in our TBI patients. Other confounding variables about PLR incudes alcohol level, smoking and so on. More importantly, in our study, we used both adjusted and non-adjusted Cox regression model as well as ROC curve to test the predicting power of PLR. All results showed that PLR is independent and powerful, which means PLR may not be significantly affected by these confounding variables at least in the case of predicting short-term mortality in patients with moderate to severe TBI.

Lymphocytes are classified into T lymphocytes, B lymphocytes and NK cells according to their origin, morphological structure, surface markers and immune function. They are important cellular components of the body's immune response. Animal experiments have shown that focal cortical contusion and subcortical neuronal damage occur within minutes after trauma, accompanied by a rapid local neuroinflammatory response^34,35^. This contributes to immune cells (macrophages, granulocytes, dendritic cells, NK cells etc.) accumulation in the injured area and surrounding tissues. The immune cell-derived cytokines (IL-1β, IL-18, TNFα, etc.) and chemokines (CCL2, CCL22, CCL17, etc.) further recruit immune cells (neutrophils, lymphocytes, lymphocytes, etc.) to the site of injury and coordinate subsequent activities^36,37^. This kind of immune response generates a self-perpetuating pro-inflammatory environment that damages the brain parenchyma. Peripheral inflammation also affects the outcome of TBI^38^. Peripheral immune cells rapidly expand and are activated after TBI with remarkable extravasation from spleen. These immune cells contribute to both innate and adaptive immune responses after TBI, with some infiltrating the central nervous system^38,39^. Once in the damaged brain area, T cells are activated by antigens on macrophages, dendritic cells, and microglia. The steady increase in T cell number and composition suggests a shift from innate non-specific immune to adaptive immune^40,41^. Decreased PLR level suggests early coagulation imbalance and neuroinflammation hyperactivity, which is independently associated with short-term mortality.

The limitations of our study are: firstly, this study is a single-center study. Secondly, this study is a retrospective cohort study. Although we use the multivariate regression model to reduce the interference, it is still unable to fully guarantee the complete balance and comparability of each group. Finally, further randomized controlled trials are needed to determine the underlying mechanism of the association between PLR and short-term mortality.

## Conclusion

PLR level is an independent biomarker with great diagnostic power for short-term mortality in patients with moderate to severe brain injury.

## Data Availability

The datasets generated and/or analyzed during the current study are not publicly available due to further study but are available from the corresponding author on reasonable request.

## Abbreviations

TBI: Traumatic brain injury
GCS: Glasgow Come Score
PLR: Platelets-to-lymphocyte ratio
NICU: Neuro-Intensive Care Unit
AIS: Abbreviate Injury Score
HR: Hazard ratios
CI: Confidence intervals
BBB: Blood–brain barrier
PD: Peritoneal dialysis
